# A randomized open-label trial on the use of budesonide/formoterol (Symbicort^®^) as an alternative reliever medication for mild to moderate asthmatic attacks

**DOI:** 10.1186/1865-1380-5-16

**Published:** 2012-04-13

**Authors:** Keng Sheng Chew, Hamizah Kamarudin, Che Wan Hashim

**Affiliations:** 1 Emergency Medicine Department, School of Medical Sciences, Health Campus, Universiti Sains Malaysia, 16150 Kota Bharu, Kelantan, Malaysia; 2 Respiratory and Medical Clinic, Kota Bharu Medical Centre, 16150 Kota Bharu, Malaysia

## Abstract

**Background:**

Conventionally, a nebulized short-acting β-2 agonist like salbutamol is often used as the reliever in acute exacerbations of asthma. However, recent worldwide respiratory outbreaks discourage routine use of nebulization. Previous studies have shown that combined budesonide/formoterol (Symbicort^®^, AstraZeneca) is effective as both a maintenance and reliever anti-asthmatic medication.

**Methods:**

We performed a randomized, open-label study from March until August 2011 to compare the bronchodilatory effects of Symbicort^® ^vs. nebulized salbutamol in acute exacerbation of mild to moderate asthmatic attack in an emergency department. Initial objective parameters measured include the oxygen saturation, peak expiratory flow rate (PEFR) and respiratory rate. During clinical reassessment, subjective parameters [i.e., Visual Analog Scale (VAS) and 5-point Likert scale of breathlessness] and the second reading of the objective parameters were measured. For the 5-point Likert scale, the patients were asked to describe their symptom relief as 1, much worse; 2, a little worse; 3, no change; 4, a little better; 5, much better.

**Results:**

Out of the total of 32 patients enrolled, 17 patients (53%) were randomized to receive nebulized salbutamol and 15 (47%) to receive Symbicort^®^. For both treatment arms, by using paired t- and Wilcoxon signed rank tests, it was shown that there were statistically significant improvements in oxygen saturation, PEFR and respiratory rate within the individual treatment groups (pre- vs. post-treatment). Comparing the effects of Symbicort^® ^vs. nebulized salbutamol, the average improvement of oxygen saturation was 1% in both treatment arms (*p *= 0.464), PEFR 78.67 l/min vs. 89.41 l/min, respectively (*p *= 0.507), and respiratory rate 2/min vs. 2/min (*p *= 0.890). For subjective evaluation, all patients reported improvement in the VAS (average 2.45 cm vs. 2.20 cm), respectively (*p *= 0.765). All patients in both treatment arms reported either "a little better" or "much better" on the 5-point Likert scale, with none reporting "no change" or getting worse.

**Conclusion:**

This study suggests that there is no statistical difference between using Symbicort^® ^vs. nebulized salbutamol as the reliever for the first 15 min post-intervention.

## Background

Defined as a chronic inflammatory airway disorder with bronchial hyper-responsiveness to a variety of stimuli, bronchial asthma is often punctuated with recurrent episodes of exacerbations with classical manifestations such as wheezing, breathlessness, chest tightness, and nocturnal or early morning cough. These episodes are usually associated with widespread but variable airflow obstruction within the lung that is often reversible either spontaneously or with treatment [1].

Conventionally, a nebulized short-acting β_2 _agonist (SABA) like salbutamol is often used as the reliever in acute exacerbations of asthma. However, the recent worldwide respiratory outbreaks, such as the H1N1 pandemic flu (in 2009) and the severe acute respiratory syndrome (SARS) outbreak (in 2002-2003), have discouraged routine nebulization because of its apparent association with air-borne infection spread [2].

Recent studies have shown that the similar bronchodilator delivered via a metered-dose inhaler, combined with a spacer, produces at least an equivalent improvement in lung function as the same dose as delivered via a nebulizer [3]. In fact, a previous study has found that with MDI and a spacer, the dose that reaches the lungs ranges from 15% to 20% of the total dose, whereas jet nebulizers deposit only 8% to 10% of the total dose [4].

Nonetheless, despite the effectiveness of delivering a bronchodilator via the metered dose route [3,4], nebulized salbutamol is still very much preferred because of its convenience [5]. Most patients when visiting an emergency department expect to be given "something more" than the metered dose inhalers that they had been taking at home.

Formoterol is a long-acting β_2_-agonist (LABA) that has a rapid onset bronchodilatory effect within 1-3 min of inhalation [6]. Its rapid bronchodilatory effect is comparable with that of salbutamol, thus making it a suitable alternative for the treatment of acute asthma and to prevent exercise-induced bronchospasm [7]. In fact, formoterol has been shown to produce greater lung improvements than salbutamol up to 4 h after administration [8].

One of the reasons why formoterol exhibits a rapid onset is because formoterol acts as a full agonist to beta-2 receptors compared to salbutamol that acts as a partial beta-2 agonist [6]. Furthermore, there are additional non-bronchodilatory beneficial effects of formoterol including the effect of reducing plasma exudation by closing the gaps between endothelial cells, stabilizing the mast cells and reducing neutrophilic recruitment, and thus reducing the release of reactive oxygen species through the activated neutrophils [9].

Combined budesonide/formoterol in a single inhaler (Symbicort^®^, AstraZeneca) has been shown to be effective as a maintenance and reliever anti-asthmatic medication [10]. In fact, this combination therapy has recently been used in an effective novel approach called Symbicort^® ^as Maintenance and Reliever Therapy (SMART) approach [9,11]. SMART approach basically means using Symbicort^® ^as a maintenance therapy twice daily with additional puffs of the combination inhaler as needed for symptom relief [9,11].

## Methods

In this randomized, open-label study conducted from March until August 2011, we compared the effects of Symbicort^® ^(at the strength of 160/4.5 mcg per inhalation) and nebulized salbutamol as the initial reliever in acute exacerbation of mild to moderate bronchial asthma in the Emergency Department, Hospital Universiti Sains Malaysia (HUSM). We defined mild and moderate asthmatic attack according to the guidelines outlined in the Global Initiative of Asthma (GINA) report on a global strategy for asthma management and prevention. Adult patients aged 18 years old and above who came to the Emergency Department of HUSM with mild to moderate asthmatic attacks were included in the study. We excluded patients referred from district hospitals or other health facilities (who may have been given reliever treatment prior to arrival), pregnancy, patients who are already started on any LABA medications as well as patients with persistent poor inhalational techniques despite being taught the proper technique in the Emergency Department. This study was approved by the hospital's ethics and research committee.

Patients who consented to participate in this study were randomized to receive either two puffs of Symbicort^® ^or nebulized salbutamol. Patients randomized to receive Symbicort^® ^were given proper instruction on the inhalational technique using the dummy turbuhaler devices supplied by AstraZeneca (Malaysia). Initial objective parameters obtained include the respiratory rate, oxygen saturation and peak expiratory flow rate (PEFR). The patients were then reassessed after 15 min. Objective and subjective variables related to the study were then recorded. Subjective parameters related to patients' perception of symptomatic relief were assessed using the Visual Analog Scale (VAS) and the 5-point Likert scale of breathlessness.

VAS is a widely used measurement scale for many symptoms including pain and breathlessness at a specific point in time. We recorded two readings (pre- and post-intervention) on a 10-cm scale with the spectrum of completely asymptomatic on one end and the most severe asthmatic symptoms ever experienced on the other end. In this study, we adopted the finding from a previous study where a change of 2.2 cm is defined as the minimal clinically significant improvement of asthma management in the emergency departments [12].

For the 5-point Likert scale, during reassessment of the patient 15 min after giving the allocated treatment, the patients were asked to describe their symptom relief on a 5-point scale of 1, much worse; 2, a little worse; 3, no change; 4, a little better; 5, much better

Subsequent management after the initial treatment and the decision for patient disposition were decided according to the discretion of the treating doctor based on the clinical improvement of the patient concerned. Patients who need admission are admitted accordingly, and patients with the nebulized salbutamol deemed fit for discharge are discharged with inhaled budesonide (Pulmicort^® ^100 mcg per inhalation, AstraZeneca) at a dose of two inhalations twice daily besides inhaled salbutamol on an as-needed basis for 30 days. Patients in the Symbicort^® ^arm are discharged with two inhalations of Symbicort^® ^as maintenance besides additional doses on an as-needed basis for up to eight doses for 30 days. All patients are given an appointment to attend a follow-up visit after 30 days to assess symptom progression.

All data were analyzed using Statistical Package for Social Sciences (SPSS^®^) Statistics software version 19.0.0. For categorical analysis, chi-square and Fisher's exact tests were used. For numerical analysis, dependent and independent Student's t-tests were used for parametric data, and Wilcoxon's signed rank test and Mann-Whitney test were used for nonparametric dependent and independent data, respectively. The block randomization process used in this study was generated using software available from the web [13].

## Results

Out of the total of 32 patients enrolled, 17 (53%) were randomized to receive nebulized salbutamol and 15 (47%) to receive Symbicort^® ^(Table 1). None of the patients required admission, and none of them required additional doses of relievers.

**Table 1:** Baseline characteristics and between-group analysis

	**Symbicort**^®^	Nebulized salbutamol	*p *value
Total no. of patients (*n*, %)	15	17	
Mean age in years (SD)	36.47 (16.26)	42.59 (15.86)	
Gender			
Male	5	9	
Female	10	8	
Objective parameters			
Mean PEFR improvement (SD) in l/min	Δ 78.67(44.86)	Δ 89.41(45.48)	0.507
Median value (IQR) of improvement of SaO_2 _in %	Δ 1 (1)	Δ 2 (1)	0.464
Median value (IQR) of reduction in respiratory rate (per min)	∇ 2 (2)/min	∇ (3)/min	0.890
Subjective parameters			
5-point Likert scale:	-	-	
1--much worse	-	-	
2--a little worse	-	-	
3--no change	-	-	
4--a little better	9 (60%)	9 (52.9%)	
5--much better	6 (40%)	8 (47.1%)	
VAS changes in cm (SD)	2.45 (1.31)	2.20 (2.10)	0.765

Note:

Δ denotes an increase in value; ∇ denotes a decrease in value

IQR means interquartile range

For patients within the Symbicort^® ^arm, by using the paired t-test for PEFR (parametric) and Wilcoxon signed rank test for respiratory rate and SaO_2 _(non-parametric), it was shown that there were statistically significant improvements in SaO_2 _and PEFR, and reduction in the respiratory rate (improvement of tachypnea) in patients after using Symbicort^® ^tubuhaler. Similar statistically significant changes were demonstrated in the nebulized salbutamol arm (Table 2).

**Table 2:** Within-group analysis for objective analysis in both arms

Intervention	Variable	Mean (SD) or median (IQR)		*p *value
		Pre-	Post-	
**Symbicort**^®^	^1^Improvement in SaO_2_	98.0 (3)%	99.0 (2)%	0.038
	^1^Reduction in respiratory rate	18 (4)/min	16 (4)/min	0.001
	^2^Improvement in PEFR	196 (88.87) l/min	274.67 (119.75) l/min	< 0.001
**Nebulized** **salbutamol**	^1^Improvement in SaO_2_	97.0 (3)%	99.0 (2)%	0.039
	^1^Reduction in respiratory rate	19 (4)/min	17 (2)/min	0.002
	^2^Improvement in PEFR	237.65 (100.59) l/min	327.06 (98.73) l/min	< 0.001

Note:

1. SaO_2 _and respiratory rate are non-parametric data in both nebulized salbutamol and Symbicort^® ^arms. The values for both pre- and post-interventions are median and interquartile range values. Wilcoxon signed rank test was used for the analysis

2. PEFR is a parametric datum. The values for both pre- and post-interventions are mean and standard deviation values. Dependent t-test was used for the analysis

Arguably, although the statistically significant changes in this study may not be noticeable clinically, yet, on subjective assessment, regardless of the type of intervention, 18 out of 32 (56%) patients reported being "a little better" on the 5-point Likert scale, and another 14 out of the 32 (44%) reported a "much better" response. All patients reported improvement in the VAS with the mean VAS of 2.45 cm and 2.20 cm in the Symbicort^® ^and nebulized salbutamol arms, respectively (Table 1).

Specifically, in the Symbicort^® ^arm, on the 5-point Likert scale, nine patients (60%) said they felt "a little better," and another six patients (40%) said they felt "much better." Similarly, for the nebulized salbutamol arm, nine patients (52.9%) felt "a little better," and eight patients (47.1%) felt "much better" on the 5-point Likert scale. None of the patients in either arm reported feeling "no change," "a little worse" or "much worse" after interventions (Table 1). Incidentally, as all patients responded as being either "a little better" or "much better," we performed a post-hoc analysis using the chi-square test to compare the subjective improvement of Symbicort^® ^versus nebulized salbutamol, and we found that there is no statistically significant difference between these two treatments (*p *= 0.688).

## Discussion

In this study, both nebulized salbutamol and Symbicort^® ^demonstrated objective and subjective clinical improvements in the first 15 min. Although not specifically designed to show non-inferiority of one drug over another, our study does suggest that Symbicort^® ^is as effective as nebulized salbutamol when used as a reliever in the emergency department for the first 15 min, which is consistent with the findings from another study that Symbicort^® ^is as effective as salbutamol in metered-dose inhalation [14].

Even though at this stage it is premature to state whether Symbicort^® ^can or should be used as an alternative reliever in acute asthmatic attacks, nonetheless, we demonstrated that both Symbicort^® ^and salbutamol offer similar improvement in objective and subjective assessments, and that there is no difference between the two.

This study is severely limited because of the inherent weaknesses of its methodology. It is an open-label, non-blinded trial involving a small sample size of 32 patients. Furthermore, in this study, the patient's comorbidities, duration of asthma and risk factors such as smoking status were not taken into consideration. However, this study may pave the way for larger studies in the future to study the beneficial effects of Symbicort^® ^beyond the first 15 min and whether it can be used as an alternative reliever. A better alternative methodology would be to add a placebo inhaler for patients in the nebulized salbutamol arm and a placebo nebulization (e.g., saline) for the Symbicort^® ^arm. In this way, the confounding bias due to the different drug delivery systems could be minimized.

Furthermore, one of the common problems in long-term asthma care is poor adherence to inhaled corticosteroid (ICS) maintenance therapy, resulting in under-treatment of the underlying airway inflammation. This is often because patients do not understand the difference between maintenance and reliever medications as well as the importance of regular ICS use [15,16]. As a result, many patients tend to over-rely on short-acting β-_2 _agonist medications for quick relief of symptoms at the expense of omitting their ICS therapy, thus lowering anti-inflammatory protection and increasing the propensity for the development of severe and potentially life-threatening exacerbations [17]. If an alternative agent that combines both reliever and maintenance medications could be used, this would simplify treatment and provide a more convenient and effective way in which to deliver medications to the endobronchial tree.

## Conclusion

In conclusion, this study shows that when Symbicort^® ^is used as a reliever in acute mild to moderate asthmatic attacks, it does not demonstrate any statistical difference when compared to nebulized salbutamol in the first 15 min. Ultimately, with the recent development of many respiratory outbreaks, we hope that the findings in this study can translate into helping to minimize the occurrence of unnecessary nebulizations by using an alternative agent that is not only equally effective, but also well accepted by the patients.

## Competing interests

The authors received dummy turbuhaler devices from AstraZeneca (Malaysia) solely for the purpose of patient education. No other support was received from AstraZeneca (Malaysia).

## Authors' contributions

KSC: involved in the initial conception, study design, data acquisition, statistical analysis, result interpretation and write-up of the manuscript. HK: contributed to the design of the study, completed the task of data acquisition as well as statistical analysis and data interpretation of the study. CWH contributed to the initial conception, design as well as intellectual contents of the study. All authors read and approved the final manuscript.

## References

[B1] GINA Global strategy for asthma management and prevention (updated 2010) 2010 http://www.ginasthma.org Accessed on 4 November 2011

[B2] Varia M Wilson S Sarwal S McGeer A Gournis E Galanis E Henry B Investigation of a nosocomial outbreak of severe acute respiratory syndrome (SARS) in Toronto, Canada CMAJ 2003 169 4 285 292 12925421 PMC180651

[B3] Cates CJ Crilly JA Rowe BH Holding chambers (spacers) versus nebulisers for betaagonist treatment of acute asthma Cochrane Database Syst Rev 2006 2 CD000052 16625527 10.1002/14651858.CD000052.pub2

[B4] Newman SP Therapeutic inhalation agents and devices. Effectiveness in asthma and bronchitis Postgrad Med 1984 76 5 194-203 206-207 6435107 10.1080/00325481.1984.11698763

[B5] Weber EJ Silverman RA Callaham ML Pollack CV Woodruff PG Clark S Camargo CA Jr A prospective multicenter study of factors associated with hospital admission among adults with acute asthma Am J Med 2002 113 5 371 378 10.1016/S0002-9343(02)01242-1 12401531

[B6] Palmqvist M Ibsen T Mellen A Lotvall J Comparison of the relative efficacy of formoterol and salmeterol in asthmatic patients Am J Respir Crit Care Med 1999 160 1 244 249 10390407 10.1164/ajrccm.160.1.9901063

[B7] Boonsawat W Charoenratanakul S Pothirat C Sawanyawisuth K Seearamroongruang T Bengtsson T Brander R Selroos O Formoterol (OXIS) Turbuhaler as a rescue therapy compared with salbutamol pMDI plus spacer in patients with acute severe asthma Respir Med 2003 97 9 1067 1074 10.1016/S0954-6111(03)00139-2 14509562

[B8] Bateman ED Fairall L Lombardi DM English R Budesonide/formoterol and formoterol provide similar rapid relief in patients with acute asthma showing refractoriness to salbutamol Respir Res 2006 7 13 10.1186/1465-9921-7-13 16433920 PMC1386666

[B9] Barnes PJ Scientific rationale for using a single inhaler for asthma control Eur Respir J 2007 29 3 587 595 10.1183/09031936.00080306 17329493

[B10] Aziz I Lipworth BJ A bolus of inhaled budesonide rapidly reverses airway subsensitivity and beta2-adrenoceptor down-regulation after regular inhaled formoterol Chest 1999 115 3 623 628 10.1378/chest.115.3.623 10084466

[B11] O'Byrne PM Bisgaard H Godard PP Pistolesi M Palmqvist M Zhu Y Ekstrom T Bateman ED Budesonide/formoterol combination therapy as both maintenance and reliever medication in asthma Am J Respir Crit Care Med 2005 171 2 129 136 15502112 10.1164/rccm.200407-884OC

[B12] Karras DJ Sammon ME Terregino CA Lopez BL Griswold SK Arnold GK Clinically meaningful changes in quantitative measures of asthma severity Acad Emerg Med 2000 7 4 327 334 10.1111/j.1553-2712.2000.tb02231.x 10805619

[B13] Saghaei M Random Allocation Software Randomization http://mahmoodsaghaei.tripod.com/Softwares/randalloc.html#Version Accessed: March 9, 2010 10.1186/1471-2288-4-26 PMC533876 15535880

[B14] Balanag VM Yunus F Yang PC Jorup C Efficacy and safety of budesonide/formoterol compared with salbutamol in the treatment of acute asthma Pulm Pharmacol Ther 2006 19 2 139 147 10.1016/j.pupt.2005.04.009 16009588

[B15] Haughney J Barnes G Partridge M Cleland J The Living & Breathing Study: a study of patients' views of asthma and its treatment Prim Care Respir J 2004 13 1 28 35 10.1016/j.pcrj.2003.11.007 16701634 PMC6750659

[B16] FitzGerald JM Boulet LP McIvor RA Zimmerman S Chapman KR Asthma control in Canada remains suboptimal: the Reality of Asthma Control (TRAC) study Can Respir J 2006 13 5 253 259 16896426 10.1155/2006/753083 PMC2683303

[B17] Kaplan A Ryan D The role of budesonide/formoterol for maintenance and relief in the management of asthma Pulm Pharmacol Ther 2010 23 2 88 96 10.1016/j.pupt.2009.10.011 19878732

